# The Impact of Sarcopenia Onset Prior to Cancer Diagnosis on Cancer Survival: A National Population-Based Cohort Study Using Propensity Score Matching

**DOI:** 10.3390/nu15051247

**Published:** 2023-03-01

**Authors:** Chih-Hsiung Su, Wan-Ming Chen, Ming-Chih Chen, Ben-Chang Shia, Szu-Yuan Wu

**Affiliations:** 1 Department of Accounting Information, Chihlee University of Technology, Taipei 22050, Taiwan; 2 Graduate Institute of Business Administration, College of Management, Fu Jen Catholic University, Taipei 24205, Taiwan; 3 Artificial Intelligence Development Center, Fu Jen Catholic University, Taipei 24205, Taiwan; 4 Department of Food Nutrition and Health Biotechnology, College of Medical and Health Science, Asia University, Taichung 41354, Taiwan; 5 Division of Radiation Oncology, Lo-Hsu Medical Foundation, Lotung Poh-Ai Hospital, Yilan 265501, Taiwan; 6 Big Data Center, Lo-Hsu Medical Foundation, Lotung Poh-Ai Hospital, Yilan 265501, Taiwan; 7 Department of Healthcare Administration, College of Medical and Health Science, Asia University, Taichung 41354, Taiwan; 8 Cancer Center, Lo-Hsu Medical Foundation, Lotung Poh-Ai Hospital, Yilan 265501, Taiwan; 9 Centers for Regional Anesthesia and Pain Medicine, Taipei Municipal Wan Fang Hospital, Taipei Medical University, Taipei 110301, Taiwan; 10 Department of Management, College of Management, Fo Guang University, Yilan 265501, Taiwan

**Keywords:** sarcopenia, nonsarcopenia, cancers, survival, prognosis

## Abstract

Purpose: The relationship between the onset of sarcopenia prior to cancer diagnosis and survival outcomes in various types of cancer is not well understood. To address this gap in knowledge, we conducted a propensity score-matched population-based cohort study to compare the overall survival of cancer patients with and without sarcopenia. Patients and Methods: In our study, we included patients with cancer and divided them into two groups based on the presence or absence of sarcopenia. To ensure comparability between the groups, we matched patients in both groups at a ratio of 1:1. Results: After the matching process, our final cohort included 20,416 patients with cancer (10,208 in each group) who were eligible for further analysis. There were no significant differences between the sarcopenia and nonsarcopenia groups in terms of confounding factors such as age (mean 61.05 years versus 62.17 years), gender (52.56% versus 52.16% male, 47.44% versus 47.84% female), comorbidities, and cancer stages. In our multivariate Cox regression analysis, we found that the adjusted hazard ratio (aHR; 95% confidence interval [CI]) of all-cause death for the sarcopenia group compared to the nonsarcopenia group was 1.49 (1.43–1.55; *p* < 0.001). Additionally, the aHRs (95% CIs) of all-cause death for those aged 66–75, 76–85, and >85 years (compared to those aged ≤65 years) were 1.29 (1.23–1.36), 2.00 (1.89–2.12), and 3.26 (2.97–3.59), respectively. The aHR (95% CI) of all-cause death for those with a Charlson comorbidity index (CCI) ≥ 1 compared to those with a CCI of 0 was 1.34 (1.28–1.40). The aHR (95% CI) of all-cause death for men compared to women was 1.56 (1.50–1.62). When comparing the sarcopenia and nonsarcopenia groups, the aHRs (95% CIs) for lung, liver, colorectal, breast, prostate, oral, pancreatic, stomach, ovarian, and other cancers were significantly higher. Conclusion: Our findings suggest that the onset of sarcopenia prior to cancer diagnosis may be linked to reduced survival outcomes in cancer patients.

## 1. Introduction

Sarcopenia is a condition marked by the reduction of muscle mass, strength, and physical performance [1,2]. It is generally defined as a decrease in appendicular muscle mass by two standard deviations below the mean for young, healthy adults [3]. Unlike cachexia, sarcopenia does not necessarily result from an underlying illness [4]. However, many patients with cachexia also have sarcopenia, while most people with sarcopenia do not have cachexia [4]. Sarcopenia is linked to higher rates of functional impairment, disability, falls, and death [5]. The causes of sarcopenia are complex and may include muscle disuse, changes in endocrine function, chronic diseases, inflammation, insulin resistance, and nutritional deficiencies [1]. Therefore, sarcopenia is distinct from cachexia and may occur before cancer develops.

Sarcopenia has a range of causes, including changes in endocrine function, proinflammatory cytokine activation, decreased alpha motor neurons in the spinal cord, reduced physical activity, and insufficient protein intake [6,7,8,9,10,11]. Research on the relationship between sarcopenia and cancer outcomes has produced conflicting results, with some studies showing an association between sarcopenia and poor cancer outcomes, while others have found no association [12,13,14,15,16]. These inconsistencies may be due to the inclusion of various cancer types, different definitions of sarcopenia (occurring before cancer diagnosis, related to cancer, or related to cancer treatment), and insufficient follow-up time in the studies [12,13,14,15,16]. Additionally, the measurement of oncological outcomes varies among studies [12,13,14,15,16].

The impact of sarcopenia on long-term survival appears to be significant across a broad range of cancer types. Sarcopenia diagnosis before a cancer diagnosis is crucial to differentiate cancer-related sarcopenia from cancer-treatment-induced sarcopenia. In this study, we used a head-to-head propensity score matching (PSM) approach including patients with cancer with and without sarcopenia to determine the oncological outcome of overall survival (OS) in these patients.

## 2. Materials and Methods

### 2.1. Study Cohort

For this study, we obtained data on patients with and without sarcopenia from the Taiwan Cancer Registry database (TCRD). These patients received a cancer diagnosis between 1 January 2008 and 31 December 2017, with the index date being the date of a cancer diagnosis. The follow-up period for these patients extended from the index date to 31 December 2019. The study protocols were reviewed and approved by the Institutional Review Board of Tzu-Chi Medical Foundation. In addition to the cancer registry database, we also used data from the Collaboration Center of Health Information Application, which provided additional information on cancer type, stage, and treatment for each patient [17]. We also tracked the vital status and cause of death of each patient.

### 2.2. Patients Selection

To be included in this study, patients had to be over the age of 20 and have a diagnosis of cancer without metastasis. We defined cancer patients as those with primary cancer. Patients with a history of cancer before the primary cancer diagnosis date (index data) were excluded from the study. The TCRD was used to verify the accuracy of all enrolled patients with primary cancer. Additionally, patients with synchronous or metachronous cancers were excluded from the cohort. To ensure that we included adult patients at risk of cancer, we defined our study population as those aged 20 years or older in Taiwan. Additionally, cancer patients with metastasis can have different survival outcomes depending on the extent of metastasis. Therefore, to avoid bias, we excluded patients with cancer and metastasis. Sarcopenia diagnosis was made before the cancer diagnosis date, and patients who did not have sarcopenia before the cancer diagnosis date were included as controls.

Sarcopenia is a muscle disease that results from adverse muscle changes that accumulate over a person’s lifetime. It is a common condition among older adults, but it can also occur earlier in life. The EWGSOP2 consensus paper defines sarcopenia as having low muscle strength as a key characteristic [18]. The diagnosis of sarcopenia is confirmed by detecting low muscle quantity and quality, while poor physical performance is indicative of severe sarcopenia. Therefore, sarcopenia was defined according to a previous study from the NHIRD [19] and was only recorded if it was diagnosed by rehabilitation specialists, orthopedics, or family physicians based on EWGSOP2 consensus [18].

The previous study employed the following protocol to define sarcopenia [19]: before 2016, there was no consensus on the definition of sarcopenia, and a variety of diagnostic criteria were being used [20]. In October 2016, the U.S. Centers for Disease Control and Prevention formally recognized sarcopenia as a disease, coding it as M62.84 in ICD-10-CM [21]. In general, the sarcopenia-related ICD-9-CM codes 728.2 and 728.9 can be considered equivalent to the ICD-10-CM code M62.84 [22]. The criteria have been used by other studies and are considered as similar to the diagnosis of sarcopenia [22]. In addition, the diagnosis of the sarcopenia-related ICD-9-CM codes 728.2 and 728.9 and ICD-10-CM code M62.84 were all verified by professional specialists (such as rehabilitation, orthopedic, or family physician). We defined the sarcopenia group in our study as “sarcopenia, muscular wasting, disuse atrophy, and disorder”.

### 2.3. Covariates and Propensity Score Matching

To analyze the time from the index date to all-cause death for patients with cancer with and without sarcopenia, we used a time-dependent Cox proportional hazards model that was adjusted for potential confounders. To account for potential confounders when comparing all-cause death between the sarcopenia and nonsarcopenia groups, we used propensity score matching. The variables used for matching included age, sex, Charlson comorbidity index (CCI) score, diabetes, hyperlipidemia, hypertension, end-stage renal disease (ESRD), liver cirrhosis, acute myocardial infarction (AMI), coronary artery disease (CAD), stroke, hepatitis B and C, congestive heart failure, dementia, chronic pulmonary disease, rheumatic disease, liver disease, diabetes with complications, hemiplegia and paraplegia, renal disease, acquired immune deficiency syndrome (AIDS), cancer type, cancer stage, income levels, and urbanization (see Table 1). We excluded repeat comorbidities from the CCI scores to prevent repetitive adjustment in the multivariate analysis. Cancer stages in our study were based on the clinical American Joint Committee on Cancer, Seventh Edition, which divides cancer types into early (stages I-II) and advanced (stages III-IV, with metastases removed) stages.

Comorbidities were identified based on ICD-9-CM or ICD-10-CM codes in the main diagnosis of inpatient records or if the patient had at least two outpatient visits within one year. Comorbidities that were present six months before the index date were recorded. Continuous variables are presented as the mean ± standard deviation or median (first quartile and third quartile), as appropriate. To match participants at a ratio of 1:1, we used the greedy method and matched participants with a propensity score within a caliper of 0.2 [23] based on the aforementioned covariates. Matching is a common technique for selecting controls with similar background characteristics to study participants in order to minimize differences between the two groups. We used a Cox model to perform the regression analysis of all-cause death in patients with cancer with and without sarcopenia and employed a robust sandwich estimator to account for clustering within the matched sets [24]. A multivariate Cox regression analysis was performed to calculate hazard ratios with 95% confidence intervals (CIs) in order to identify potential independent predictors of all-cause death among the variables listed in Table 1.

### 2.4. Sensitivity Analysis

**Figure 1:**
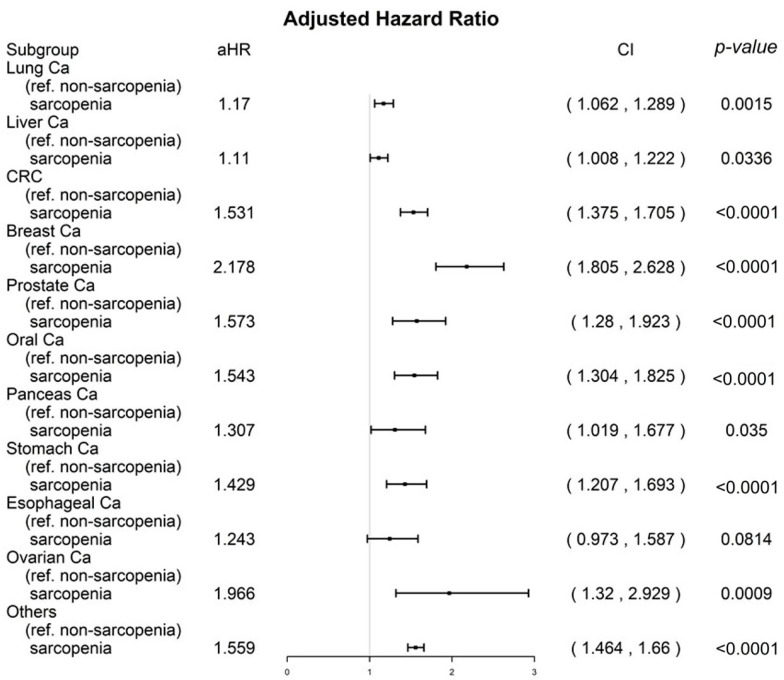
A sensitivity analysis of different cancer types by using inverse probability of treatment weighting for all-cause death in the propensity score–matched sarcopenia and nonsarcopenia groups. All covariates presented in Table 2 were adjusted.

To understand the relationship between mortality and sarcopenia in patients with various types of cancer, a sensitivity analysis was conducted using inverse probability of treatment weighting (IPTW) for all-cause death in propensity score-matched sarcopenia and nonsarcopenia groups. The analysis adjusted for covariates listed in Table 2 and included all cancer types (as shown in Figure 1).

### 2.5. Statistical Analysis

The statistical analyses for this study were conducted using SAS version 9.4 (SAS Institute, Cary, NC, USA). The matching procedure was implemented using the PROC PSMATCH procedure in SAS [25]. A two-tailed Wald test was used, and a *p* value of less than 0.05 was considered statistically significant. Overall survival (OS) was estimated using the Kaplan–Meier method and the differences in OS between the sarcopenia and nonsarcopenia groups with cancer were determined using the stratified log-rank test to compare survival curves, stratified according to the matched sets [26].

## 3. Results

### 3.1. Study Cohort

There was a total of 103,925 cancer patients included in the registry during the selected time frame. Before PSM, out of all the cancer patients included in the registry, 14.9% were diagnosed with sarcopenia prior to their cancer diagnosis. This means that there were 15,527 sarcopenic cancer patients out of the total of 103,925 cancer patients, while the remaining 88,398 were nonsarcopenic cancer patients. Therefore, the percentage of sarcopenic patients out of all cancer patients is approximately 14.9%.

Propensity score matching resulted in a final study cohort of 20,416 patients, with 10,208 in both the sarcopenia and nonsarcopenia groups. The characteristics of these patients are listed in Table 1. The age distribution was balanced between the two groups (Table 1). Additionally, after head-to-head PSM, there were no significant differences in sex distribution, CCI score, diabetes, hyperlipidemia, hypertension, ESRD, liver cirrhosis, AMI, CAD, stroke, hepatitis B and C, congestive heart failure, dementia, chronic pulmonary disease, rheumatic disease, liver disease, diabetes with complications (severe diabetes), hemiplegia and paraplegia, renal disease, AIDS, cancer type, cancer stage, income levels, and urbanization between the two groups. The primary endpoint of all-cause death in the sarcopenia group (before cancer diagnosis) was significantly different from the nonsarcopenia group (*p* < 0.001; Table 1).

### 3.2. Multivariate Cox Regression Analysis

The results of the multivariate Cox regression analysis indicated that patients with cancer and sarcopenia before cancer diagnosis had a shorter OS compared to those without sarcopenia (see Table 2). The adjusted hazard ratio (aHR; 95% CI) of all-cause mortality for the sarcopenia group compared to the nonsarcopenia group was 1.49 (1.43 to 1.55; *p* < 0.001). Several explanatory variables were found to be significantly associated with an increased risk of all-cause mortality. These included older age, being male, and having a CCI score of 1 or higher. Specifically, the aHRs (95% CIs) of all-cause mortality for those aged 66 to 75, 76 to 85, and over 85 years (compared to those aged 65 or younger) were 1.29 (1.23 to 1.36), 2.00 (1.89 to 2.12), and 3.26 (2.97 to 3.59), respectively (see Table 2). The aHR (95% CI) of all-cause mortality for those with a CCI score of 1 or higher compared to those with a CCI score of 0 was 1.34 (1.28 to 1.40). The aHR (95% CI) of all-cause mortality for men compared to women was 1.56 (1.50 to 1.62). No other explanatory variables were found to be significantly associated with an increased risk of all-cause mortality.

### 3.3. Sensitivity Analysis for Cancer Types

A stratified analysis based on IPTW was conducted to examine the distinct age groups and CCI scores, and the results are presented in a forest plot in Figure 1. Among patients with lung, liver, colorectal, breast, prostate, oral, pancreatic, stomach, ovarian, and other types of cancer, the adjusted hazard ratios (aHRs; 95% confidence intervals [CIs]) for all-cause mortality in the sarcopenia group were 1.17 (1.06 to 1.29), 1.11 (1.01 to 1.22), 1.53 (1.38 to 1.71), 2.18 (1.81 to 2.63), 1.57 (1.28 to 1.92), 1.54 (1.30 to 1.83), 1.31 (1.02 to 1.68), 1.43 (1.21 to 1.69), 1.97 (1.32 to 2.93), and 1.56 (1.46 to 1.66), respectively. These aHRs were significantly associated with higher mortality in the sarcopenia group compared to the nonsarcopenia group, regardless of the age group, sex, or CCI score range (as shown in Figure 1). In addition, the aHR (95% CI) for all-cause mortality for patients with esophageal cancer was 1.24 (0.97 to 1.59, *p* = 0.0814) in the sarcopenia group compared to the nonsarcopenia group.

### 3.4. Sensitivity Analysis of CCI Score, Age Groups, and Sex

**Figure 2:**
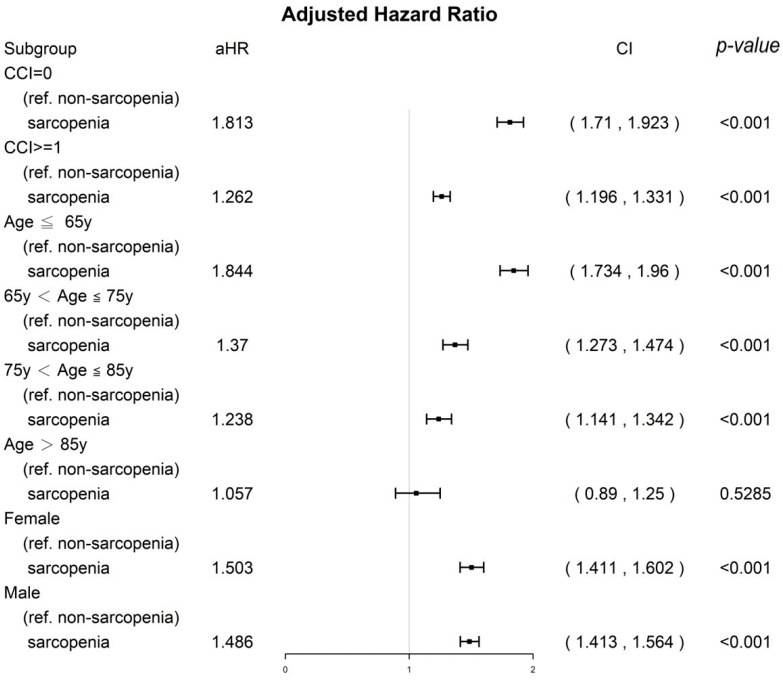
A sensitivity analysis of sex, age groups, and CCI scores by using inverse probability of treatment weighting for all-cause death in the propensity score–matched sarcopenia and nonsarcopenia groups. All covariates presented in Table 2 were adjusted.

The results of the stratified analysis of the distinct age groups and CCI scores based on IPTW are presented as a forest plot in Figure 2. Among patients with cancer, the aHRs (95% CIs) for all-cause mortality for the sarcopenia group were 1.81 (1.71 to 1.92) for those with a CCI score of 0, 1.26 (1.20 to 1.33) for those with a CCI score of 1 or higher, 1.49 (1.41 to 1.56) for men, 1.50 (1.41 to 1.60) for women, 1.84 (1.73 to 1.96) for those aged 65 or younger, 1.37 (1.27 to 1.47) for those aged 66 to 75, and 1.24 (1.14 to 1.34) for those aged 76 to 85. These aHRs were significantly associated with higher mortality in the sarcopenia group compared to the nonsarcopenia group, regardless of the cancer type (as shown in Figure 2). Poor OS in relatively healthy individuals (those with a CCI score of 0), female sex, and younger age group (65 or younger) were more significant in the sarcopenia group than in the nonsarcopenia group.

### 3.5. Kaplan–Meier Survival Curves

**Figure 3:**
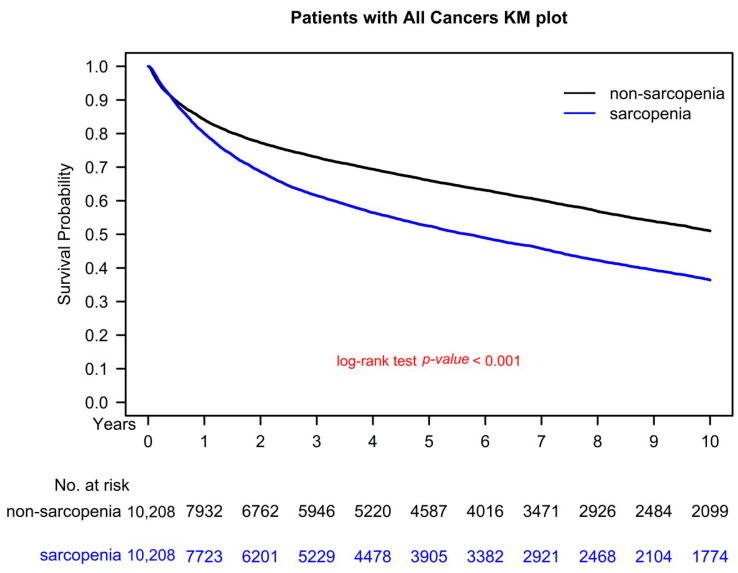
Kaplan–Meier overall survival curves for the propensity score–matched sarcopenia and nonsarcopenia groups (controls).

Figure 3 presents the survival curve (in terms of OS) for the propensity score–matched sarcopenia (diagnosed with sarcopenia before cancer treatment) and nonsarcopenia groups, calculated using the Kaplan–Meier method. The 5-year OS for individuals who did not use opioids was 69.97%, while the 5-year OS for those who used long-term opioid analgesics was 51.82% (*p* < 0.001). This difference in OS between the two groups was statistically significant.

## 4. Discussion

Cachexia is different from sarcopenia, which refers to the loss of skeletal muscle mass that is often two SDs below values that are adjusted for sex and age [27]. Most of the individuals with cachexia have sarcopenia, whereas most of the patients with sarcopenia do not have cachexia [27]. Muscle loss without fat loss is known as sarcopenic obesity, which is prevalent in older adults and is noted in patients with advanced cancer [28,29,30]. Sarcopenia can have various causes, including disuse atrophy, changes in endocrine function, chronic diseases, inflammation, insulin resistance, nutritional deficiencies, and certain cancer treatments such as sorafenib and androgen deprivation [6,7,8,9,10,11,31,32,33]. To investigate the effect of sarcopenia as a predictor of OS on patients with cancer, we included only patients who were diagnosed as having sarcopenia before cancer diagnosis to exclude cancer-related or cachexia-related sarcopenia and cancer-treatment-related sarcopenia. In this study, we aimed to investigate the impact of sarcopenia on the overall survival of cancer patients. To the best of our knowledge, our study has the largest sample size and the longest follow-up period compared to other studies that have examined the relationship between OS and sarcopenia in cancer patients.

Sarcopenia has been shown to be a significant predictor of survival in various types of cancer [12]. Sarcopenia has gained attention in the field of oncology due to its potential impact on cancer prognosis and the associated financial strain it can place on individuals and society [34]. Sarcopenia is a hallmark of cachexia [35]. Sarcopenia in cancer patients has been linked to reduced tolerance to anticancer treatment, increased susceptibility to cancer-treatment-related complications such as infection and immobility, and an increased risk of comorbidities [31]. These factors can contribute to higher mortality rates in cancer patients with sarcopenia compared to those without sarcopenia [31]. According to one study, reversing muscle wasting in a cancer cachexia model was found to improve survival outcomes. Additionally, a randomized controlled trial (RCT) showed that pharmacological agents can be effective in increasing muscle mass in cancer cachexia [36,37]. Therefore, it is crucial to acknowledge that sarcopenia can be modified in cancer patients. Our results indicated that sarcopenia onset before cancer is a poor prognostic factor for OS (Table 2 and Figure 1, Figure 2 and Figure 3); hence, early detection and treatment of sarcopenia are crucial and might be associated with improved OS in patients with cancer in the future. To determine whether reversing sarcopenia before cancer diagnosis could improve the OS of cancer patients, an RCT is necessary.

Several previous meta-analyses have indicated that there is a link between sarcopenia and increased mortality in cancer patients [38,39,40]. However, it is not clear whether the findings from these meta-analyses, which have mostly focused on specific types of cancer, can be generalized to a wider range of cancer types [38,39,40]. The survival effect of sarcopenia diagnosis before cancer diagnosis in different cancer types remains largely unclear. Furthermore, multiple endpoints have been noted in previous studies that contributed to heterogeneous outcomes [12,13,14,15,16] and a comparative long-term study on the survival of head-to-head PSM sarcopenia and nonsarcopenia groups is still lacking. In addition, insufficient sample sizes have been used in comparative studies for sarcopenia and nonsarcopenia in a wide spectrum of cancer types for OS outcomes. Our study has the largest sample size among studies examining the survival effect of sarcopenia in patients with a wide spectrum of cancer. Moreover, our mean follow-up time for the sarcopenia and nonsarcopenia groups including the patients with cancer was >5 years; this follow-up period was sufficient to evaluate survival outcomes (Table 1).

All confounding factors associated with mortality were balanced between the sarcopenia and nonsarcopenia groups receiving cancer treatment (Table 1). Cancer types and clinical cancer stages were homogenized between the sarcopenia and nonsarcopenia groups through PSM to evaluate the true survival effect of sarcopenia on patients with cancer because patients with different cancer types and cancer stages have different survival durations. As shown in Table 1, the crude all-cause death rate after PSM was significantly higher in the sarcopenia group than in the nonsarcopenia group. The head-to-head PSM design allows for an observational (nonrandomized) study approach that is similar to a randomized controlled trial (RCT) in some ways [41]. After PSM in our study, we believe that balanced covariates mimic an RCT without selection bias for the sarcopenia and nonsarcopenia groups [41]. According to the results of multivariate Cox proportional analysis (as shown in Table 2), the onset of sarcopenia before cancer diagnosis was found to be an independent predictor of all-cause mortality in cancer patients. Our literature review showed that our study had the largest sample size, longest follow-up period, and widest range of cancer types of any study using PSM to investigate whether the onset of sarcopenia before a cancer diagnosis is a significant predictor of OS in cancer patients. Figure 3 presents the results of this comparative study. Additionally, the results of the multivariate analysis showed that old age, a CCI of 1 or higher, and being male were poor prognostic factors for OS, as seen in Table 2. This finding is consistent with the results of previous studies on various types of cancer [42,43].

Our sensitivity analysis of a broad range of cancer types (including the top 10 most common cancers in Taiwan) found that sarcopenia significantly increased the risk of all-cause mortality in patients with lung, liver, colorectal, breast, prostate, oral, pancreatic, stomach, ovarian, and other types of cancer (as shown in Figure 1). Our study demonstrated that sarcopenia onset before a cancer diagnosis is an independent predictor of OS. Our findings are partially consistent with those of previous studies that have examined specific cancer types and used ill-defined definitions of sarcopenia, including sarcopenia that occurs before, after, or during cancer diagnosis [12,13,14,15,16]. One of the disadvantages of using an ill-defined definition of the time interval for sarcopenia (such as before, after, or during cancer diagnosis) is that the conclusions may be biased if the presence of cancer-related cachexia or cancer treatment-induced sarcopenia is not taken into account, rather than noncancer-related sarcopenia. Cancer-related cachexia or cancer-treatment-induced sarcopenia is different from sarcopenia prior to cancer diagnosis because the mechanisms are different for noncancer sarcopenia, cancer-related sarcopenia, and cancer-treatment-related sarcopenia [6,7,8,9,10,11,31,32,33]. Unlike cachexia, sarcopenia does not necessarily involve weight loss [28,29,30]. Noncancer-related sarcopenia can be caused by a range of factors, including disuse, changes in endocrine function, nutritional deficiencies, chronic diseases, inflammation, and insulin resistance [6,7,8,9,10,11]. Cancer-induced or cancer-treatment-induced sarcopenia may be attributed to cachexia or anticancer treatments in patients with cancer [6,7,8,9,10,11,28,29,30,31,32,33]. Toxicity caused by cancer-related-inflammation-induced cachexia and cancer treatments might be contributed to cancer-related sarcopenia instead of noncancer sarcopenia [6,7,8,9,10,11,28,29,30,31,32,33]. Our study focused on noncancer sarcopenia diagnosed prior to cancer diagnosis, and we excluded cancer-related sarcopenia to avoid the bias of different cancer types or different treatments.

After conducting a sensitivity analysis of the CCI, sex, and age (which were identified as significant independent factors for OS in cancer patients in Table 2), we found that a diagnosis of sarcopenia prior to cancer diagnosis remained a significantly poor prognostic factor for OS (as shown in Figure 2), regardless of CCI score, sex, or age group. However, sarcopenia did not have a significant effect on OS in cancer patients who were over the age of 85. Moreover, in sensitivity analysis, the aHR of OS was lower in men, patients with CCI ≥ 1, and older patients. This finding might be because the patients with cancer with a significantly high risk of mortality, such as those with CCI ≥ 1, male patients, and older patients (Table 2), had a shorter life expectancy than did those with CCI = 0, female patients, or younger patients. Therefore, the survival effect of sarcopenia might be masked by patients with cancer with a shorter life expectancy (CCI ≥ 1, male sex, and old age), contributing to decreased aHRs (Figure 2). Therefore, a diagnosis of sarcopenia prior to cancer diagnosis was not a significant prognostic factor for OS in cancer patients who were older (over the age of 85) and had a relatively short life expectancy (as shown in Figure 2).

One of the strengths of our study is that it is the first, largest, and longest-term follow-up comparative cohort study to examine the primary endpoint of OS in cancer patients with and without sarcopenia. To eliminate selection bias, PSM was used to ensure that the covariates between the two groups were homogenous for cancer patients (as seen in Table 1). This is the first study to investigate this relationship in such a comprehensive and in-depth manner. Studies estimating the survival effect of sarcopenia onset before cancer diagnosis on all-cause death in a wide spectrum of cancer types are rare. In our study, we found that poor prognostic factors for OS in cancer patients included the onset of sarcopenia before cancer diagnosis, high CCIs, being male, and being of advanced age (as seen in Table 2 and Figure 2 and Figure 3). These findings are consistent with those of previous cancer studies [42,43]. Our findings suggest that the onset of sarcopenia before cancer diagnosis may be associated with poorer OS in patients with lung, liver, colorectal, breast, prostate, oral, pancreatic, stomach, ovarian, and other types of cancer, compared to those without sarcopenia (as shown in Figure 1). To our knowledge, this is the first study to report that the impact of sarcopenia onset prior to cancer diagnosis on survival was stronger in cancer patients with a longer life expectancy, such as those with breast, prostate, or colorectal cancer, a CCI of 0, female patients, or younger patients (as seen in Figure 1 and Figure 2). In the patients with cancer with a longer life expectancy, a higher aHR of sarcopenia for mortality was noted in patients with cancer than in those with cancer but without sarcopenia. By contrast, the survival effect of sarcopenia was lower and masked, especially in the patients with cancer with a shorter life expectancy such as those with liver cancer, esophageal cancer, pancreatic cancer, CCI ≥ 1, male patients, or older patients (Figure 1 and Figure 2). Previous studies have not specifically focused on the impact of sarcopenia onset before cancer diagnosis in a wide range of cancer types. Our study is the first to examine the effects of this factor on all-cause mortality and in a diverse group of cancer types. Our findings should be taken into account in future clinical practice and prospective clinical trials to prevent or treat sarcopenia onset before cancer treatment, particularly in relatively healthy patients (those with a CCI of 0), women, younger patients, and those with cancer types that have a longer survival prognosis (as shown in Figure 1 and Figure 2).

This study has a few limitations that should be noted. Firstly, as all the patients enrolled in the study were Asian, it is unclear whether these results apply to non-Asian populations. However, there is no evidence to suggest that there are significant differences in oncological outcomes between Asian and non-Asian cancer survivors. Secondly, the diagnoses of all comorbid conditions were based on ICD-9-CM or ICD-10-CM codes. While the Taiwan Cancer Registry Administration regularly reviews medical charts and interviews patients to verify the accuracy of diagnoses, a large-scale randomized trial comparing carefully selected patients with sarcopenia onset prior to cancer diagnosis and those without sarcopenia would be necessary to gain more specific information on the population characteristics and disease occurrence. However, it is important to note that these measures do not completely eliminate the possibility of error in diagnosis, and hospitals with outlier charges or practices may be audited and face penalties if malpractice or discrepancies are identified. Finally, it is worth noting that the Taiwan Cancer Registry database does not include data on dietary habits or body mass index, which may be risk factors for OS. Despite this limitation, the study has several strengths, including the use of a nationwide population-based registry with detailed baseline information and the ability to conduct long-term follow-up through the linkage of the registry with the national Cause of Death database. The observed effects in this study were both statistically significant and of a large magnitude, indicating that they are unlikely to be affected by these limitations.

## 5. Conclusions

Our findings suggest that the onset of sarcopenia before cancer diagnosis may be associated with a reduction in OS and poorer OS in patients with lung, liver, colorectal, breast, prostate, oral, pancreatic, stomach, ovarian, and other types of cancer compared to those without sarcopenia. The impact of sarcopenia onset prior to cancer diagnosis on survival was stronger in patients with a longer life expectancy, such as those with breast, prostate, or colorectal cancer, a CCI of 0, female patients, or younger patients.

## Figures and Tables

**Table 1:** Characteristics of patients with cancer in the sarcopenia and nonsarcopenia groups receiving cancer treatments (after propensity score matching).

	Nonsarcopenia		Sarcopenia		SMD
	N = 10,208		N = 10,208		
	N	%	N	%	
**Age (mean ± SD)**	61.05 ± 15.78		62.17 ± 14.28		0.075
**Age (years)**					0.031
Age ≤ 65	5584	54.70	5620	55.05	
65 < Age ≤ 75	2573	25.21	2496	24.45	
75 < Age ≤ 85	1729	16.94	1719	16.84	
Age > 85	322	3.15	373	3.65	
**Sex**					0.008
Female	4843	47.44	4883	47.84	
Male	5365	52.56	5325	52.16	
**Diabetes**	2085	20.43	2100	20.57	0.004
**Hypertension**	4388	42.99	4345	42.56	0.009
**Hyperlipidemia**	2212	21.67	2254	22.08	0.010
**ESRD**	133	1.30	127	1.24	0.005
**Liver cirrhosis**	2370	23.22	2433	23.83	0.015
**AMI**	180	1.76	209	2.05	0.021
**Coronary arterial disease**	2114	20.71	2178	21.34	0.015
**Stroke**	582	5.70	652	6.39	0.029
**Hepatitis C**	317	3.11	329	3.22	0.002
**Hepatitis B**	581	5.69	574	5.62	0.003
**CCI score (mean ± SD)**	0.91 ± 1.24		0.93 ± 1.27		0.018
**CCI score**					0.005
=0	5613	54.99	5589	54.75	
≥1	4595	45.01	4619	45.25	
**CCI**					
Congestive heart failure	549	5.38	552	5.41	0.001
Dementia	208	2.04	229	2.24	0.014
Chronic pulmonary disease	1889	18.51	2019	19.78	0.032
Rheumatic disease	133	1.30	154	1.51	0.017
Liver disease	2173	21.29	2228	21.83	0.013
Diabetes mellitus with complications	479	4.69	465	4.56	0.007
Hemiplegia and paraplegia	0		0		-
Renal disease	590	5.78	593	5.81	0.001
AIDS	5	0.05	4	0.04	0.005
**Income levels**					0.009
Low income	96	0.94	104	1.02	0.001
Income ≤20,000 NTD/month	6305	61.77	6284	61.56	
20,000 < income ≤ 30,000 NTD/month	2370	23.22	2377	23.29	
Income > 30,000 NTD/month	1437	14.08	1443	14.14	
**Urbanization**					0.001
Rural	3209	31.44	3204	31.39	
Urban	6999	68.56	7004	68.61	
**Cancer types**					
**Lung cancer**	1474	14.44	1474	14.44	0.000
Early stages	678	6.64	678	6.64	
Advanced stages	796	7.80	796	7.80	
**Hepatocellular carcinoma**	1174	11.50	1174	11.50	0.000
Early stages	698	6.84	698	6.84	
Advanced stages	476	4.66	476	4.66	
**Colorectal cancer**	1501	14.70	1501	14.70	0.000
Early stages	911	8.92	911	8.92	
Advanced stages	590	5.78	590	5.78	
**Breast cancer**	1004	9.84	1004	9.84	0.000
Early stages	502	4.92	502	4.92	
Advanced stages	502	4.92	502	4.92	
**Prostate cancer**	396	3.88	396	3.88	0.000
Early stages	132	1.29	132	1.29	
Advanced stages	264	2.59	264	2.59	
**Head and neck cancer**	592	5.80	592	5.80	0.000
Early stages	269	2.64	269	2.64	
Advanced stages	323	3.16	323	3.16	
**Pancreatic cancer**	232	2.27	232	2.27	0.000
Early stages	106	1.04	106	1.04	
Advanced stages	126	1.23	126	1.23	
**Gastric cancer**	503	4.93	503	4.93	0.000
Early stages	240	2.35	240	2.35	
Advanced stages	263	2.58	263	2.58	
**Esophagus cancer**	252	2.47	252	2.47	0.000
Early stages	42	0.41	42	0.41	
Advanced stages	210	2.06	210	2.06	
**Ovarian cancer**	164	1.61	164	1.61	0.000
Early stages	66	0.65	66	0.65	
Advanced stages	98	0.96	98	0.96	
**Other cancers**	4115	40.31	4115	40.31	0.000
Early stages	2244	21.98	2244	21.98	
Advanced stages	1871	18.33	1871	18.33	
					***p* value**
**Follow-up, years (mean ± SD)**	6.49 ± 4.82		5.88 ± 4.63		<0.0001
**Follow-up, years; median (IQR, Q1,Q3)**	5.17 (1.19, 8.82)		4.16 (1.04, 7.80)		<0.0001
**All-cause death**					<0.0001
No	5920	57.99	4306	42.18	
Yes	4288	42.01	5902	57.82	

AIDS, acquired immune deficiency syndrome; AMI, acute myocardial infarction; CCI, Charlson comorbidity index; ESRD, end-stage renal disease; IQR, interquartile range; SD, standard deviation; SMD, standardized mean difference; NTD, New Taiwan Dollars.

**Table 2:** Univariable and multivariable Cox proportional regression model for all-cause death of the propensity score–matched sarcopenia and nonsarcopenia groups.

	Crude HR (95% CI)		Adjusted HR * (95% CI)		*p* Value
**Sarcopenia** (ref.: Nonsarcopenia)					
Yes	1.5	(1.44, 1.56)	1.49	(1.43, 1.55)	<0.001
**Sex** (ref.: Female)					
Male	1.74	(1.67, 1.81)	1.56	(1.50, 1.62)	<0.001
**Age** (years; ref.: Age ≤ 65)					
65 < Age ≤ 75	1.64	(1.57, 1.72)	1.29	(1.23, 1.36)	<0.001
75 < Age ≤ 85	2.75	(2.61, 2.89)	2.00	(1.89, 2.12)	<0.001
Age > 85	4.64	(4.24, 5.08)	3.26	(2.97, 3.59)	<0.001
**CCI score** (ref. = 0)					
≥1	1.82	(1.75, 1.89)	1.34	(1.28, 1.40)	<0.001
**Diabetes** (ref.: No)					
Yes	1.17	(1.02, 1.78)	1.06	(0.89, 1.32)	0.357
**Hyperlipidemia** (ref.: No)					
Yes	1.14	(1.08, 1.41)	1.08	(0.93, 1.03)	0.446
**Liver cirrhosis** (ref.: No)					
Yes	1.11	(1.05, 1.38)	1.02	(0.91, 1.22)	0.275
**Hypertension** (ref.: No)					
Yes	1.25	(1.18, 1.82)	1.04	(0.79, 1.20)	0.428
**ESRD** (ref.: No)					
Yes	1.19	(1.05, 1.96)	1.07	(0.88, 1.60)	0.131
**AMI** (ref.: No)					
Yes	1.47	(0.83, 2.34)	1.12	(0.85, 1.36)	0.226
**Coronary arterial disease** (ref.: No)					
Yes	1.66	(1.58, 1.74)	1.09	(0.74, 1.50)	0.390
**Stroke** (ref.: No)					
Yes	1.23	(1.07, 2.39)	1.17	(0.98, 1.37)	0.071
**Hepatitis C** (ref.: No)					
Yes	1.88	(1.17, 2.08)	1.06	(0.95, 1.18)	0.284
**Hepatitis B** (ref.: No)					
Yes	1.67	(0.93, 1.68)	1.11	(0.88, 1.81)	0.393
**Urbanization** (ref.: Rural)					
Urban	0.87	(0.83, 0.9)	0.94	(0.90, 1.02)	0.196
**Income** (ref.: Low income)					
Income ≤ 20,000 NTD/month	0.78	(0.65, 0.94)	0.88	(0.73, 1.06)	0.179
20,000 < Income ≤ 30,000 NTD/month	0.68	(0.56, 0.82)	0.82	(0.68, 1.09)	0.155
Income > 30,000 NTD/month	0.39	(0.32, 0.48)	0.61	(0.50, 1.14)	0.101

HR, hazard ratio; AMI, Acute myocardial infarction; CCI, Charlson comorbidity index; CI, confidence interval; ESRD, end-stage renal disease; ref., reference group; NTD, New Taiwan Dollars; * All covariates presented in Table 2 were adjusted.

## Data Availability

The datasets supporting the study conclusions are included within this manuscript.

## Abbreviations

aHR: adjusted hazard ratio; CI, confidence interval; RCT, randomized controlled trial; PSM, propensity score matching; ICD-9-CM, International Classification of Diseases, Ninth Revision, Clinical Modification; ICD-10-CM, International Classification of Diseases, Tenth Revision, Clinical Modification; ESRD, end-stage renal disease; AMI, acute myocardial infarction; CAD, coronary artery disease; AIDS, acquired immune deficiency syndrome; OS, overall survival; CCI, Charlson comorbidity index; IPTW, inverse probability of treatment weighting.

## References

[1] Janssen I. (2011). The epidemiology of sarcopenia. Clin. Geriatr. Med..

[2] Cruz-Jentoft A.J., Baeyens J.P., Bauer J.M., Boirie Y., Cederholm T., Landi F., Martin F.C., Michel J.P., Rolland Y., Schneider S.M. (2010). Sarcopenia: European consensus on definition and diagnosis: Report of the European Working Group on Sarcopenia in Older People. Age Ageing.

[3] Baumgartner R.N., Waters D.L., Gallagher D., Morley J.E., Garry P.J. (1999). Predictors of skeletal muscle mass in elderly men and women. Mech. Ageing Dev..

[4] Muscaritoli M., Anker S.D., Argiles J., Aversa Z., Bauer J.M., Biolo G., Boirie Y., Bosaeus I., Cederholm T., Costelli P. (2010). Consensus definition of sarcopenia, cachexia and pre-cachexia: Joint document elaborated by Special Interest Groups (SIG) “cachexia-anorexia in chronic wasting diseases” and “nutrition in geriatrics”. Clin. Nutr..

[5] Janssen I. (2006). Influence of sarcopenia on the development of physical disability: The Cardiovascular Health Study. J. Am. Geriatr. Soc..

[6] Houston D.K., Tooze J.A., Garcia K., Visser M., Rubin S., Harris T.B., Newman A.B., Kritchevsky S.B., Health A.B.C.S. (2017). Protein Intake and Mobility Limitation in Community-Dwelling Older Adults: The Health ABC Study. J. Am. Geriatr. Soc..

[7] Kyle U.G., Morabia A., Schutz Y., Pichard C. (2004). Sedentarism affects body fat mass index and fat-free mass index in adults aged 18 to 98 years. Nutrition.

[8] Rasmussen B.B., Fujita S., Wolfe R.R., Mittendorfer B., Roy M., Rowe V.L., Volpi E. (2006). Insulin resistance of muscle protein metabolism in aging. FASEB J..

[9] Joseph C., Kenny A.M., Taxel P., Lorenzo J.A., Duque G., Kuchel G.A. (2005). Role of endocrine-immune dysregulation in osteoporosis, sarcopenia, frailty and fracture risk. Mol. Aspects Med..

[10] Roberts S.B. (2000). Regulation of energy intake in relation to metabolic state and nutritional status. Eur. J. Clin. Nutr..

[11] Szulc P., Duboeuf F., Marchand F., Delmas P.D. (2004). Hormonal and lifestyle determinants of appendicular skeletal muscle mass in men: The MINOS study. Am. J. Clin. Nutr..

[12] Li H.L., Au P.C., Lee G.K., Li G.H., Chan M., Cheung B.M., Wong I.C., Lee V.H., Mok J., Yip B.H. (2021). Different definition of sarcopenia and mortality in cancer: A meta-analysis. Osteoporos. Sarcopenia.

[13] Au P.C., Li H.L., Lee G.K., Li G.H., Chan M., Cheung B.M., Wong I.C., Lee V.H., Mok J., Yip B.H. (2021). Sarcopenia and mortality in cancer: A meta-analysis. Osteoporos. Sarcopenia.

[14] Ibilibor C., Psutka S.P., Herrera J., Rivero J.R., Wang H., Farrell A.M., Liss M.A., Pruthi D., Mansour A.M., Svatek R. (2021). The association between sarcopenia and bladder cancer-specific mortality and all-cause mortality after radical cystectomy: A systematic review and meta-analysis. Arab. J. Urol..

[15] Zhang X.M., Dou Q.L., Zeng Y., Yang Y., Cheng A.S.K., Zhang W.W. (2020). Sarcopenia as a predictor of mortality in women with breast cancer: A meta-analysis and systematic review. BMC Cancer.

[16] Joglekar S., Nau P.N., Mezhir J.J. (2015). The impact of sarcopenia on survival and complications in surgical oncology: A review of the current literature. J. Surg. Oncol..

[17] Zhang J., Lu C.Y., Chen H.M., Wu S.Y. (2021). Neoadjuvant Chemotherapy or Endocrine Therapy for Invasive Ductal Carcinoma of the Breast with High Hormone Receptor Positivity and Human Epidermal Growth Factor Receptor 2 Negativity. JAMA Netw. Open.

[18] Cruz-Jentoft A.J., Bahat G., Bauer J., Boirie Y., Bruyere O., Cederholm T., Cooper C., Landi F., Rolland Y., Sayer A.A. (2019). Sarcopenia: Revised European consensus on definition and diagnosis. Age Ageing.

[19] Sun M.Y., Chang C.L., Lu C.Y., Wu S.Y., Zhang J.Q. (2022). Sarcopenia as an Independent Risk Factor for Specific Cancers: A Propensity Score-Matched Asian Population-Based Cohort Study. Nutrients.

[20] Bijlsma A.Y., Meskers C.G., Ling C.H., Narici M., Kurrle S.E., Cameron I.D., Westendorp R.G., Maier A.B. (2013). Defining sarcopenia: The impact of different diagnostic criteria on the prevalence of sarcopenia in a large middle aged cohort. Age.

[21] Anker S.D., Morley J.E., von Haehling S. (2016). Welcome to the ICD-10 code for sarcopenia. J. Cachexia Sarcopenia Muscle.

[22] Lin M.H., Chiu S.Y., Chang P.H., Lai Y.L., Chen P.C., Ho W.C. (2020). Hyperlipidemia and Statins Use for the Risk of New Diagnosed Sarcopenia in Patients with Chronic Kidney: A Population-Based Study. Int. J. Environ. Res. Public Health.

[23] Austin P.C. (2011). Optimal caliper widths for propensity-score matching when estimating differences in means and differences in proportions in observational studies. Pharm. Stat..

[24] Austin P.C. (2013). The performance of different propensity score methods for estimating marginal hazard ratios. Stat. Med..

[25] Yuan Y., Yung Y.-F., Stokes M. Propensity Score Methods for Causal Inference with the PSMATCH Procedure. Proceedings of the SAS Global Forum 2017 Conference.

[26] Austin P.C. (2014). The use of propensity score methods with survival or time-to-event outcomes: Reporting measures of effect similar to those used in randomized experiments. Stat. Med..

[27] Baumgartner R.N., Koehler K.M., Gallagher D., Romero L., Heymsfield S.B., Ross R.R., Garry P.J., Lindeman R.D. (1998). Epidemiology of sarcopenia among the elderly in New Mexico. Am. J. Epidemiol..

[28] Kim T.N., Yang S.J., Yoo H.J., Lim K.I., Kang H.J., Song W., Seo J.A., Kim S.G., Kim N.H., Baik S.H. (2009). Prevalence of sarcopenia and sarcopenic obesity in Korean adults: The Korean sarcopenic obesity study. Int. J. Obes..

[29] Rolland Y., Lauwers-Cances V., Cristini C., Abellan van Kan G., Janssen I., Morley J.E., Vellas B. (2009). Difficulties with physical function associated with obesity, sarcopenia, and sarcopenic-obesity in community-dwelling elderly women: The EPIDOS (EPIDemiologie de l’OSteoporose) Study. Am. J. Clin. Nutr..

[30] Prado C.M., Lieffers J.R., McCargar L.J., Reiman T., Sawyer M.B., Martin L., Baracos V.E. (2008). Prevalence and clinical implications of sarcopenic obesity in patients with solid tumours of the respiratory and gastrointestinal tracts: A population-based study. Lancet Oncol..

[31] Antoun S., Baracos V.E., Birdsell L., Escudier B., Sawyer M.B. (2010). Low body mass index and sarcopenia associated with dose-limiting toxicity of sorafenib in patients with renal cell carcinoma. Ann. Oncol..

[32] Alibhai S.M., Breunis H., Timilshina N., Johnston C., Tomlinson G., Tannock I., Krahn M., Fleshner N.E., Warde P., Canning S.D. (2010). Impact of androgen-deprivation therapy on physical function and quality of life in men with nonmetastatic prostate cancer. J. Clin. Oncol. Off. J. Am. Soc. Clin. Oncol..

[33] Smith M.R., Saad F., Egerdie B., Sieber P.R., Tammela T.L., Ke C., Leder B.Z., Goessl C. (2012). Sarcopenia during androgen-deprivation therapy for prostate cancer. J. Clin. Oncol. Off. J. Am. Soc. Clin. Oncol..

[34] Van Vugt J.L.A., Buettner S., Levolger S., Coebergh van den Braak R.R.J., Suker M., Gaspersz M.P., de Bruin R.W.F., Verhoef C., van Eijck C.H.C., Bossche N. (2017). Low skeletal muscle mass is associated with increased hospital expenditure in patients undergoing cancer surgery of the alimentary tract. PLoS ONE.

[35] Hilmi M., Jouinot A., Burns R., Pigneur F., Mounier R., Gondin J., Neuzillet C., Goldwasser F. (2019). Body composition and sarcopenia: The next-generation of personalized oncology and pharmacology?. Pharmacol. Ther..

[36] Zhou X., Wang J.L., Lu J., Song Y., Kwak K.S., Jiao Q., Rosenfeld R., Chen Q., Boone T., Simonet W.S. (2010). Reversal of cancer cachexia and muscle wasting by ActRIIB antagonism leads to prolonged survival. Cell.

[37] Advani S.M., Advani P.G., VonVille H.M., Jafri S.H. (2018). Pharmacological management of cachexia in adult cancer patients: A systematic review of clinical trials. BMC Cancer.

[38] Shachar S.S., Williams G.R., Muss H.B., Nishijima T.F. (2016). Prognostic value of sarcopenia in adults with solid tumours: A meta-analysis and systematic review. Eur. J. Cancer.

[39] Buentzel J., Heinz J., Bleckmann A., Bauer C., Rover C., Bohnenberger H., Saha S., Hinterthaner M., Baraki H., Kutschka I. (2019). Sarcopenia as Prognostic Factor in Lung Cancer Patients: A Systematic Review and Meta-analysis. Anticancer Res..

[40] Hua X., Liu S., Liao J.F., Wen W., Long Z.Q., Lu Z.J., Guo L., Lin H.X. (2020). When the loss costs too much: A systematic review and meta-analysis of sarcopenia in head and neck cancer. Front. Oncol..

[41] Austin P.C. (2011). An Introduction to Propensity Score Methods for Reducing the Effects of Confounding in Observational Studies. Multivar. Behav. Res..

[42] Wu S.Y., Fang S.C., Shih H.J., Wen Y.C., Shao Y.H.J. (2019). Mortality associated with statins in men with advanced prostate cancer treated with androgen deprivation therapy. Eur. J. Cancer.

[43] Lin Y.K., Hsieh M.C., Wang W.W., Lin Y.C., Chang W.W., Chang C.L., Cheng Y.F., Wu S.Y. (2018). Outcomes of adjuvant treatments for resectable intrahepatic cholangiocarcinoma: Chemotherapy alone, sequential chemoradiotherapy, or concurrent chemoradiotherapy. Radiother. Oncol..

